# PSYCHOSOCIAL AND NEIGHBORHOOD-RELATED HEALTH FACTORS FOR BLACK AND WHITE ADULTS OF THE HANDLSLEEP PROJECT

**DOI:** 10.1093/geroni/igae098.0245

**Published:** 2024-12-31

**Authors:** Alyssa Gamaldo, Orfeu Buxton

**Affiliations:** Chair; Discussant

## Abstract

Disparities in cognition and sleep have been observed in middle-aged and older Black and White adults. However, limited research has examined the association between cognition and sleep as well as the underlying psychosocial, contextual, and biomarker factors that explain this association. This symposium will include presentations using data from Healthy Aging in Neighborhood of Diversity Across the Life Span Sleep Study (HANDLSleep), which was conducted to explore psychosocial and contextual factors related to sleep and cognition in a sample of Black and White middle-aged and older adults in Baltimore, MD. The objectives of the proposed symposium are the following: (1) discuss the associations observed between sleep and cognition using cross-sectional and ambulatory data (2) discuss how psychosocial, contextual, and inflammatory markers relate to sleep and cognition. Master and colleagues explored the daily coupling associations between actigraphic sleep and working memory. Allan and colleagues explored the association between different dimensions of health (e.g., sleep and cognition) and objective and subjective measures of neighborhood quality. Linying and colleagues examined the extent that mean and variability of actigraphic sleep relate to global mental status. Lastly, Shen and colleagues explored the association between actigraphic sleep and inflammatory markers and the extent this association varies by race and sex.

